# Enhancing solar spectrum utilization in photosynthesis: exploring exciton and site energy shifts as key mechanisms

**DOI:** 10.1038/s41598-023-49729-3

**Published:** 2023-12-15

**Authors:** Kõu Timpmann, Margus Rätsep, Arvi Freiberg

**Affiliations:** 1 Institute of Physics, University of Tartu, W. Ostwaldi 1, 50411 Tartu, Estonia; 2 Estonian Academy of Sciences, Kohtu 6, 10130 Tallinn, Estonia

**Keywords:** Chemical physics, Biological physics

## Abstract

Photosynthesis is a critical process that harnesses solar energy to sustain life across Earth's intricate ecosystems. Central to this phenomenon is nuanced adaptation to a spectrum spanning approximately from 300 nm to nearly 1100 nm of solar irradiation, a trait enabling plants, algae, and phototrophic bacteria to flourish in their respective ecological niches. While the Sun’s thermal radiance and the Earth’s atmospheric translucence naturally constrain the ultraviolet extent of this range, a comprehension of how to optimize the utilization of near-infrared light has remained an enduring pursuit. This study unveils the remarkable capacity of the bacteriochlorophyll *b*-containing purple photosynthetic bacterium *Blastochloris viridis* to harness solar energy at extreme long wavelengths, a property attributed to a synergistic interplay of exciton and site energy shift mechanisms. Understanding the unique native adaptation mechanisms offers promising prospects for advancing sustainable energy technologies of solar energy conversion.

## Introduction

Photosynthetic organisms, including plants, algae, and some bacteria utilize solar energy to convert inorganic matter into organic compounds. This vital process is facilitated by specialized light-harvesting (LH) chromoprotein units enriched by pigment chromophores. The absorbance of plant and algal LH chromoproteins, sensitive mainly to visible part of solar spectrum, is very distinct from this of phototrophic bacteria, which are designed to enhance the absorbance of near-infrared light not used by former species. An obvious way to accomplish this goal would be by employing pigments of different chemical nature such as chlorophylls (mostly Chl *a/b*) in plants and bacteriochlorophylls (BChl *a/b*) in bacteria. However, this naive site energy shift approach—as we call it—is not what we see in nature. Indeed, while the transition wavelengths of critical to function^1^ lowest-energy Q_y_ singlet absorption bands in Chl *a* and BChl *a* differ by about 100 nm^2^, the spectra of the same pigments arranged in native photosystems almost triple their diversion. Frequently, this vast expansion, due mostly to bacterial systems, is implicitly assigned to collective pigment excitations called excitons^3^. Tuning of light-harvesting spectra in the most basic exciton model framework can be understood as a combined effect of the interactions between transition densities of the pigments (a cause of the exciton shift component), and between pigments and their intermediate protein surroundings (a source of the site energy shift). While the concept of photosynthetic excitons is generally acknowledged, their effectiveness in elucidating spectral shifts in light harvesting has encountered constraints in both theoretical and experimental contexts. These limitations can be attributed to two primary factors. First, the size of photosynthetic chromoproteins remains impractical for comprehensive modeling^4,5^. Secondly, the scarcity of native photosystems hinders the distinct separation of site energy and exciton shift mechanisms, preventing a more in-depth understanding of the phenomenon.

To clarify this issue, in this work, the light-harvesting 1 (LH1)—reaction center (RC) super-complexes (also known as core complexes) from a number of purple photosynthetic bacteria that contain either BChl *a* or BChl *b* pigments as the major light-harvesting agents are in parallel investigated. The samples selected hold similar structure, whereby cyclic LH1 complexes of modular edifice embrace central RC complexes, see^6–8^ for reviews. The varying number of LH1 modules comprise similar αβ-BChl_2_ heterodimers of membrane-spanning α-helical α- and β-apoproteins that noncovalently bind two BChl *a* or *b* molecules. The closely coupled LH1 pigment chromophores are known to support excitons upon the photon absorption^3^.

**Figure 1:**
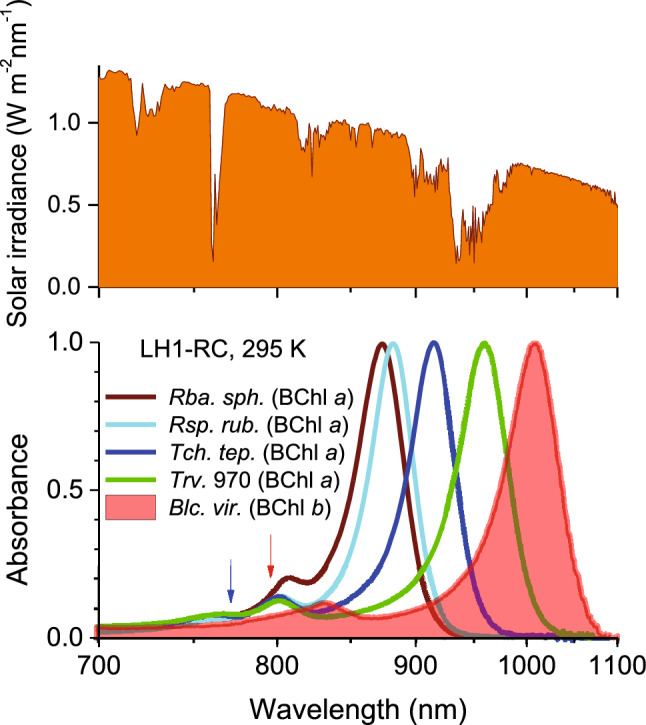
Near-infrared optical absorption spectra recorded at ambient temperature of the purified LH1-RC core complexes from different BChl *a-* and BChl *b*-containing purple bacteria, as indicated in the insertion. The most red-shifted spectrum of *Blc*. *viridis* is highlighted by red color. Blue and red arrows designate the positions of Q_y_ absorption peaks of in vitro BChl *a* and BChl *b* molecules, respectively. Shown on top is the related solar power distribution at sea level (data credit: https://www.nrel.gov/grid/solar-resource/spectra-am1.5.html). Note the linear in energy reciprocal wavelength scale commonly applied in this work.

The near-infrared absorption spectra of LH1-RC core complexes shown in Fig. 1 are dominated by LH1 complexes. A contribution of RCs seen around 750–850 nm is relatively weak. Most noteworthy, however, is that the LH1 spectra universally display a strong bathochromic (red-) shift with respect to the spectra of individual pigments (designated by arrows in Fig. 1). One should also notice that the spectra of four representative LH1s that contain BChl *a* cover greater part of the spectral range utilized by bacterial photosynthesis. While different numbers of LH1 modules doesn’t seem to play any role in this behavior, the analyses provided in^9^ considered excitons mostly responsible for the prominent spectral flexibility of the BChl *a-*containing bacteria.

The basic question now arises whether the same mechanism holds in case of the BChl *b*-containing *Blastochloris (Blc*.) *viridis*, the species that harnesses the record low-energy light among all known photosynthetic organisms. Previous attempts to solve this problem have provided incompatible results. One disturbing factor in relation with the LH1 complex of *Blc. viridis* is that it comprises a third transmembrane polypeptide, assigned as the γ subunit, which binds between the β polypeptides on the exterior of LH1. The resulting tighter packing of BChls was assumed to mostly increase excitonic couplings between pigments^10^. Experiments with deleted γ subunits indeed showed a 46 nm blue-shift in the spectrum, confirming the importance of this subunit in spectral tuning^11^. Yet the more recent electronic structure calculations^12^ and Raman spectroscopy studies^13^ emphasize that dynamic pigment macrocycle ring distortions and hydrogen bonding strength (which both translate as site energy effects) rather than exciton effects may mainly contribute into the altered *Blc*. *viridis* absorbance. As common in exciton theory, the critical to function lowest Q_y_ excitation energy of individual pigments is here briefly named as site energy. These and a number of other studies (see^5,7^ for recent reviews) reveal fundamental deficiencies in our understanding of the bases of color tuning of photosynthetic spectra.

In this research, exciton and site energy spectral shift components in BChl *b*-containing *Blc. viridis* were first quantitatively assessed in comparison with those for BChl *a*-containing core complexes. Combining fluorescence anisotropy excitation and hole-burning spectroscopies^14–16^, a unique interplay between the two mechanisms is demonstrated in the strive to extend the spectral range of photosynthetic light-harvesting into the infrared region, where more than 50% of the solar energy spectrum is distributed^17^.

## Results and discussion

**Figure 2:**
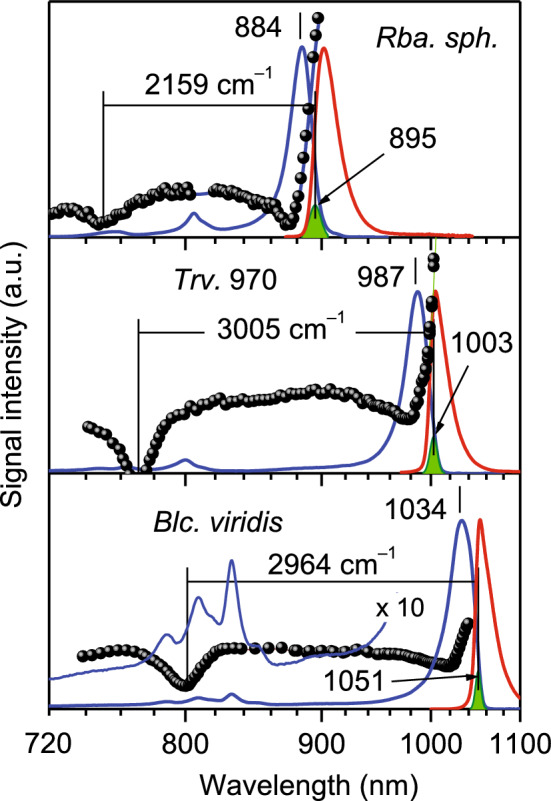
Absorption (blue), fluorescence (red), fluorescence anisotropy excitation (black balls), and hole-burning (green shapes) spectra of LH1-RC complexes from *Blc. viridis, Trv*. 970, and *Rba. sphaeroides* recorded at 4.5 K. The arbitrarily normalized spectra are shown in separate frames. The tail region of the *Blc. viridis* spectrum is amplified to display the structured RC absorption part. Numbers label spectral positions of the Q_y_ exciton absorption bands and the maximums of hole-burning action spectra (pointed by arrows) in nanometers. Horizontal lines measure exciton bandwidths in wavenumber units.

**Figure 3:**
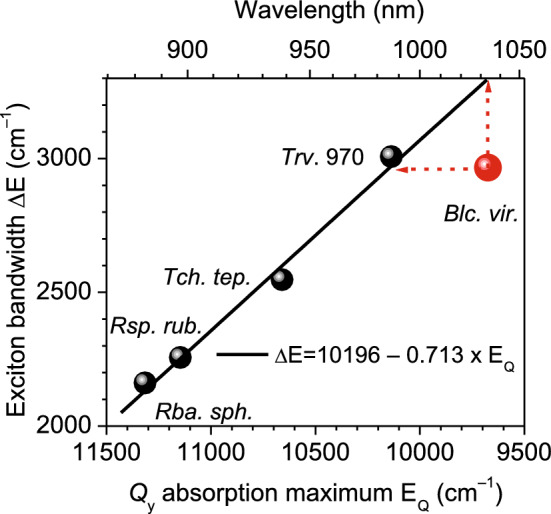
Exciton bandwidths ∆E with an estimated uncertainty of ± 20 cm^–1^ determined at 4.5 K as a function of the Q_y_ transition energy E_Q_. Data for BChl *a*-containing complexes are presented by black balls. Shown by solid line is a linear regression fit of these data.

Most of the spectroscopic measurements in this work were performed at cryogenic temperatures of 4.5 K. Presented in Fig. 2 are all the relevant experimental spectra (absorption, fluorescence, polarized fluorescence excitation, and hole-burning) for LH1-RC complexes from BChl *b*-containing *Blc. viridis* sideways with those from *Rhodobacter (Rba.) sphaeroides* and *Thiorhodovibrio* strain 970 (*Trv*. 970). The latter complexes are brought up for reference, as the complexes containing BChl *a* with the least (*Rba. sphaeroides*) and most (*Trv*. 970) red-shifted absorption spectra, respectively. The processed data for all studied complexes is shown in Fig. 3.

In the absorption spectra of Fig. 2, the LH1 and RC spectral components are well separated from each other. In *Blc. viridis*, for example, LH1 is responsible for the main asymmetric band peaking at 1034 nm, while a group of weaker bands peaking at 788, 808, 819, 832, and 851 nm can be firmly associated with RC^18,19^. The asymmetric shape of the LH1 spectrum is a result of the manifold of overlapping exciton states in the cyclic assembly of closely coupled BChl pigments^20^. The origin of a broad bump observed at 894 nm will in detail be discussed in a separate publication. Here we only indicate that it is due to coupling of LH1 excitons with high-frequency (≥ 1500 cm^–1^) BChl *b* vibrations. The spectra of reference complexes show comparable structure, albeit with shifted towards blue positions of the relevant spectral bands. Worth noticing also is that while the relative shifts of the RC bands in BChl *a*- and BChl *b*-containing bacteria roughly correspond to the Q_y_ transition energy difference between the *a* and *b* modifications of the BChl pigments, the much greater relative shift of the LH1 bands does not allow such straightforward explanation.

As initially demonstrated in reference^21^, crucial data for assessing the exciton bandwidth, denoted as ∆E, in cyclic LH complexes can be acquired through simultaneous measurements of fluorescence anisotropy excitation and hole-burning spectra. ∆E is then operationally determined as the energy difference between the high-energy dip of the polarized fluorescence excitation spectrum (which arises exciton states at the top of the exciton band) and the peak of the hole-burning action spectrum, a measure of the average position of the lowest-energy exciton state^22^. Horizontal lines in Fig. 2 indicate the so-defined exciton bandwidths. It is worth noticing that the exciton states designating exciton band edges are optically very weak, restricted by circular symmetry of the LH1 complex and near in-plane orientation of the Q_y_ transition dipole moment vectors of the pigments.

Even a brief inspection of the data in Fig. 2 leads to an important qualitative conclusion that the extreme red-shifted absorption observed in *Blc. viridis* cannot be a direct consequence of an increased exciton coupling. This is because the measured ∆E in *Blc. viridis* (2964 cm^–1^) is narrower, not broader compared with that in *Trv*. 970 (3005 cm^–1^), while its absorption spectrum is by 47 nm (461 cm^–1^) more shifted towards red.

∆E as a function of the Q_y_ exciton transition frequency, E_Q_, for all the samples analyzed in this work are shown in Fig. 3. As seen, a fine linear correlation is established between the data of BChl *a-*containing core complexes described by an equation of ∆E(E_Q_) = 10,196−0.713 × E_Q_. Yet the data associated with *Blc. viridis* clearly departs from this path. An enforced matching would require either considering much wider exciton bandwidth (∆E ≈ 3300 cm^–1^) or a greater value of E_Q_ around 10,143 cm^–1^ (~ 986 nm in wavelength scale). These two options are indicated in Fig. 3 by dashed arrows. The former assumption can be right away dismissed by present measurements which fix the width of the exciton band in *Blc. viridis*. The latter idea is, however, worth further consideration by virtue of noticing that the energy gap between the projected (986 nm) and experimental (1034 nm) absorption bands amounting 472 cm^–1^ is within the 15% error margin the same as the separation between the Q_y_ absorption bands of BChl *a* (at 771 nm) and BChl* b* (at 796 nm) dissolved in diethyl ether^23^.

The observed robust linear correlation between ∆E and E_Q_ within complexes containing BChl *a* strongly implies a dominating mechanism driving the absorption band shift in LH1 complexes. Remarkably, this mechanism appears to remain quite resilient in the face of numerous explicit variations elucidated in recent cryo-electron microscopy studies on bacterial core complexes as reported in references^6–8^. These variations encompass not only aspects such as BChl-protein hydrogen bonding and BChl conformations, which have been discussed previously, but also extend to specific binding sites for protein pigments, the presence of metal ions, and variations in carotenoid content. The celebrated property of delocalized excitons, known for their ability to mitigate the impact of various static and dynamic perturbations (as noted in reference^24^), leads us to postulate that, given the reasonably comparable structures of all studied complexes, the behavior of excitons in a hypothetical LH1 complex of *Blc. viridis*, where all BChl *b* molecules are replaced with BChl *a* molecules, would closely align with the trend depicted by the black line in Fig. 3.

What makes the present dataset truly distinctive is its capacity to disentangle, for the first time, the contributions of pigment substitution (BChl *a* to BChl b) and excitons in the red-shifted spectral response of any native photosynthetic system. In quantitative terms, the width of the exciton band, represented by ∆E = 2964 cm^–1^, implies a nearest-neighbor exciton coupling energy of approximately 740 cm^–1^ and an exciton shift value of around 1480 cm^–1^. Importantly, this shift is proportionally much larger than the estimated site energy shift of 472 cm^–1^. Unfortunately, achieving a more precise separation of these conditionally distinct spectral tuning elements remains elusive at the current empirical stage of study due to the unresolved exciton displacement shift component as discussed in reference^4^.

**Figure 4:**
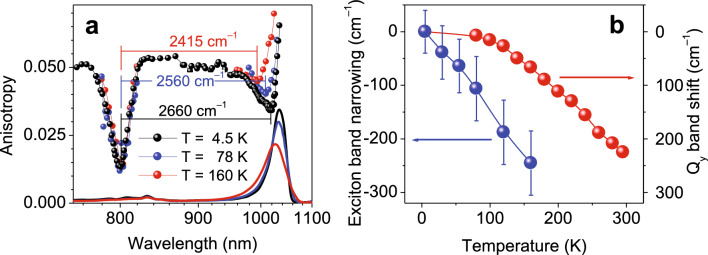
(**a**) Fluorescence anisotropy excitation (scattered data) and absorption (solid line) spectra of LH1-RC complexes from *Blc. viridis* recorded at indicated temperatures. (**b**) Temperature dependence of the Q_y_ exciton transition energy (red balls) and exciton bandwidth (blue balls). The data are plotted relative to the values recorded at 4.5 K. The lines connecting discrete data point are for leading the eye. Please also notice that differently from Fig. 2, the exciton bandwidth is here defined as the energy difference between the two dips in anisotropy spectra, see text for further explanations.

A systematic divergence might have been noticed between the position’s E_Q_ of the Q_y_ exciton absorption spectra of the LH1 complexes recorded at cryogenic and ambient temperatures, whereby the latter spectra appear at shorter wavelengths. To understand the origin of this phenomenon in case of *Blc. viridis*, temperature dependences of its absorption and fluorescence anisotropy excitation spectra were additionally studied (Fig. 4).

The data in Fig. 4a show narrowing of the exciton bandwidth with the temperature increase, in rough correlation with blue-shifting of the absorption spectra. Since the high-energy anisotropy dip maintains its position at all temperatures, the changes of the low-energy anisotropy dip and the absorption spectrum maximum are expected to more or less precisely follow each other. As seen in Fig. 4b, however, considerable discrepancies exist between the apparent rates and magnitudes of the ∆E and E_Q_ changes. This likely is an artifact caused by asymmetric modification of the exciton absorption band shape, which due to thermally-induced symmetry braking mostly concerns blue side of the Q_y_ band, exaggerating the apparent ∆E change. We note in passing that excitons in BChl *a-*containing LH1 complexes reveal similar behavior^16^. An assessment of the low-energy edge of the exciton bandwidth by hole-burning spectra, as in Fig. 2, would most probably have reduced (if not eliminated) this apparent problem. Unfortunately, this more appropriate approach is not practical here, because the narrow zero-phonon lines the technique relies on exist in photosynthetic chromoproteins only at rather low temperatures, below about 50 K^25,26^.

In summary, this study delves into the mechanisms underlying the remarkable adaptability of the purple photosynthetic bacterium *Blc. viridis*, which exhibits the extraordinary ability to function effectively at extremely long near-infrared wavelengths. This exceptional feat, which pushes the boundaries of photosynthetic activity, can be attributed to the combined effects of exciton and site energy shift mechanisms. Phototropic bacteria that rely on BChl *a* as their primary light-absorbing pigment thrive under the near-infrared light beyond approximately 800 nm, which lies outside the absorption range of chlorophylls. The excitons associated with the BChl *a* pigment play a pivotal role in efficiently harvesting light across a substantial wavelength span from 800 to 1000 nm (as depicted in Figs. 1 and 3). However, as demonstrated in this research, extending this range beyond 1000 nm necessitates the involvement of yet another pigment (in sequence Chl *a* → BChl *a* → BChl *b*) with an even redder Q_y_ transition energy. The apparent limitation in the spectral tuning capability of BChl *a*-excitons may stem from the constraints imposed by the packing density of the protein environment.

While nature naturally expands the range of solar energy available for supporting ecosystems, harnessing this energy for practical use is a critical modern technological challenge. The insights gleaned from this study regarding the roles of exciton and site energy shifts in natural photosynthetic systems hold the potential to inspire future sustainable energy strategies and innovative device development.

## Materials and methods

### Samples

The membrane and purified LH1-RC complexes from *Blc*. *viridis*, *Rba. sphaeroides*, *Rhodospirillum rubrum*, *Thermochromatium tepidum*, and *Trv*. 970, kindly donated by different laboratories (see Acknowledgements), were prepared as described earlier^13,27–30^. The concentrated samples were stored at − 78 °C in deep freezer. Prior the use the samples were diluted with 20 mM Tris–HCl pH 8.0 buffer containing 0.05% of *n*-dodecyl β-d-maltopyranoside (DDM) detergent to avoid aggregation. To obtain transparent glassy samples at low temperatures the sample solution contained, respectively, slightly increased detergent concentration (~ 0.12%) and glycerol with a 2:1 volume ratio.

### Spectroscopy

The absorption spectra were measured with Cary 60 UV–Vis spectrophotometer (Agilent) equipped with a temperature-controlled cell holder (Quantum Northwest). The fluorescence anisotropy excitation spectra^14,31^ and hole-burning spectra^32^ were recorded by a system that comprised a 0.3-m focal length spectrograph Shamrock SR-303i equipped with a thermo-electrically cooled CCD camera DV420A-OE (both Andor) and a model 3900S Ti: sapphire laser of 0.5 cm^–1^ linewidth pumped by a Millennia Prime solid-state laser (both Spectra Physics). For low-temperature measurements PMMA plastic cuvettes (Brand) were placed into a liquid helium bath cryostat (Utreks). Temperature was measured with a precision of ± 0.5 K by a Lakeshore Cryotronics calibrated silicon diode temperature controller Model 211.

### Data analyses

Data analyses and fitting procedures were performed using the Origin 9.0 SR1 (OriginLab) software.

## Data Availability

The data presented in this study are available on request from the corresponding author.

## References

[1] Reimers JR (2016). Challenges facing an understanding of the nature of low-energy excited states in photosynthesis. Biochim. Biophys. Acta - Bioenerg..

[2] Renge I, Mauring K (2013). Spectral shift mechanisms of chlorophylls in liquids and proteins. Spectrochim. Acta A: Mol. Biomol. Spectrosc..

[3] van Amerongen H, Valkunas L, van Grondelle R (2000). Photosynthetic Excitons.

[4] Maity S, Kleinekathöfer U (2023). Recent progress in atomistic modeling of light-harvesting complexes: A mini review. Photosynth. Res..

[5] Reppert M (2023). Bioexcitons by design: How do we get there?. J. Phys. Chem. B.

[6] Gardiner AT, Nguyen-Phan TC, Cogdell RJ (2020). A comparative look at structural variation among RC–LH1 ‘core’ complexes present in anoxygenic phototrophic bacteria. Photosynth. Res..

[7] Kimura Y, Tani K, Madigan MT, Wang-Otomo Z-Y (2023). Advances in the spectroscopic and structural characterization of core light-harvesting complexes from purple phototrophic bacteria. J. Phys. Chem. B.

[8] Swainsbury-David JK, Qian P, Hitchcock A, Hunter CN (2023). The structure and assembly of reaction centre-light-harvesting 1 complexes in photosynthetic bacteria. Biosci. Rep..

[9] Timpmann K (2021). Exciton origin of color-tuning in Ca^2+^-binding photosynthetic bacteria. Int. J. Mol. Sci..

[10] Qian P, Siebert CA, Wang P, Canniffe DP, Hunter CN (2018). Cryo-EM structure of the *Blastochloris viridis* LH1–RC complex at 2.9 Å. Nature.

[11] Namoon D, Rudling NM, Canniffe DP (2022). The role of the γ subunit in the photosystem of the lowest-energy phototrophs. Biochem. J..

[12] Mondragón-Solórzano G, Sandoval-Lira J, Nochebuena J, Cisneros GA, Barroso-Flores J (2022). Electronic structure effects related to the origin of the remarkable near-infrared absorption of *Blastochloris viridis*’ light harvesting 1-reaction center complex. J. Chem. Theory Comput..

[13] Kimura Y (2021). Circular dichroism and resonance raman spectroscopies of bacteriochlorophyll b-containing LH1-RC complexes. Photosynth. Res..

[14] Timpmann K, Trinkunas G, Qian P, Hunter CN, Freiberg A (2005). Excitons in core LH1 antenna complexes of photosynthetic bacteria: Evidence for strong resonant coupling and off-diagonal disorder. Chem. Phys. Lett..

[15] Pajusalu M, Rätsep M, Trinkunas G, Freiberg A (2011). Davydov splitting of excitons in cyclic bacteriochlorophyll a nanoaggregates of bacterial light-harvesting complexes between 4.5 and 263 K. Chem. Phys. Chem..

[16] Freiberg A, Pajusalu M, Rätsep M (2013). Excitons in intact cells of photosynthetic bacteria. J. Phys. Chem. B.

[17] Kruse O, Rupprecht J, Mussgnug JH, Dismukes GC, Hankamer B (2005). Photosynthesis: A blueprint for solar energy capture and biohydrogen production technologies. Photochem. Photobiol. Sci..

[18] Breton J (1985). Orientation of the chromophores in the reaction center of *Rhodopseudomonas viridis*. Comparison of low-temperature linear dichroism spectra with a model derived from x-ray crystallography. Biochim. Biophys. Acta Bioenerg..

[19] Mikhailyuk IK, Knox PP, Paschenko VZ, Razjivin AP, Lokstein H (2006). Analysis of absorption spectra of purple bacterial reaction centers in the near infrared region by higher order derivative spectroscopy. Biophys. Chem..

[20] Hu X, Ritz T, Damjanovic A, Schulten K (1997). Pigment organization and transfer of electronic excitation in the photosynthetic unit of purple bacteria. J. Phys. Chem. B.

[21] Trinkunas G, Freiberg A (2006). A disordered polaron model for polarized fluorescence excitation spectra of LH1 and LH2 bacteriochlorophyll antenna aggregates. J. Lumin..

[22] Reddy NRS, Picorel R, Small GJ (1992). B896 and B870 components of the R*hodobacter sphaeroides* antenna: A hole burning study. J. Phys. Chem..

[23] Taniguchi M, Lindsey JS (2021). Absorption and fluorescence spectral database of chlorophylls and analogues. Photochem. Photobiol..

[24] Knapp EW (1984). Lineshapes of molecular aggregates. Exchange narrowing and intersite correlation. Chem. Phys..

[25] Pajusalu M, Rätsep M, Freiberg A (2014). Temperature dependent electron-phonon coupling in chlorin-doped impurity glass and in photosynthetic FMO protein containing bacteriochlorophyll *a*. J. Lumin..

[26] Hayes JM, Lyle PA, Small GJ (1994). A theory for the temperature dependence of hole-burned spectra. J. Phys. Chem..

[27] Tani K (2020). Cryo-EM structure of a Ca^2+^-bound photosynthetic LH1-RC complex containing multiple αβ-polypeptides. Nat. Commun..

[28] Suzuki H (2007). Purification, characterization and crystallization of the core complex from thermophilic purple sulfur bacterium *Thermochromatium tepidum*. Biochim. Biophys. Acta Bioenerg..

[29] Moskalenko A, Toropygina O, Kuznetsova N (1996). Does the B820 subcomplex of the B880 complex retain carotenoids?. Z. Naturforsch. C Biosci..

[30] Sturgis JN, Olsen JD, Robert B, Hunter CN (1997). Functions of conserved tryptophan residues of the core light-harvesting complex of *Rhodobacter sphaeroides*. Biochemistry.

[31] Timpmann K, Trinkunas G, Olsen JD, Hunter CN, Freiberg A (2004). Bandwidth of excitons in LH2 bacterial antenna chromoproteins. Chem. Phys. Lett..

[32] Rätsep M, Freiberg A (2007). Electron-phonon and vibronic couplings in the FMO bacteriochlorophyll *a* antenna complex studied by difference fluorescence line narrowing. J. Lumin..

